# Serum Dkk-1 Is Associated with Pain Intensity, Flare-Ups, and Bone Mineral Density in Non-Obese Patients with Knee Osteoarthritis: A Single-Center, Cross-Sectional Study

**DOI:** 10.3390/ijms27052216

**Published:** 2026-02-26

**Authors:** Timea-Csilla Nagy-Finna, Árpád Sólyom, János Székely, Pál-István Kikeli, Erika-Lídia Szövérfi, Hunor Lukács, Anna-Lilla Faragó, Emőke Horváth, Horațiu Popoviciu, Előd Ernő Nagy

**Affiliations:** 1 Doctoral School of Medicine and Pharmacy, I.O.S.U.D., George Emil Palade University of Medicine, Pharmacy, Science, and Technology of Târgu Mureș, 540139 Târgu Mureș, Romania; csilla.nagy-finna@umfst.ro (T.-C.N.-F.);; 2 Department M3, Rheumatology, BFK and Medical Rehabilitation, George Emil Palade University of Medicine, Pharmacy, Science and Technology of Târgu Mureș, 540139 Târgu Mureș, Romania; 3 Procardia Medical Center, Mihai Eminescu Street 3, 540043 Târgu Mureș, Romania; 4 Department M3, Orthopedics-Traumatology 2, George Emil Palade University of Medicine, Pharmacy, Science, and Technology of Târgu Mureș, 38 Gheorghe Marinescu Street, 540139 Târgu Mureș, Romania; 5 Department M1, Pathology, Faculty of Medicine, George Emil Palade University of Medicine, Pharmacy, Science, and Technology of Târgu Mureș, 38 Gheorghe Marinescu Street, 540139 Târgu Mureș, Romania; 6 Department F1, Biochemistry and Environmental Chemistry, George Emil Palade University of Medicine, Pharmacy, Sciences and Technology of Târgu Mureș, 38 Gheorghe Marinescu Street, 540139 Târgu Mureș, Romania; 7 Laboratory of Medical Analysis, Mures Clinical County Hospital, 1 Gheorghe Marinescu Street, 540103 Târgu Mureș, Romania

**Keywords:** osteoarthritis, knee, DKK-1, KOFUS, VAS

## Abstract

Osteoarthritis is the most common musculoskeletal disorder. It primarily affects people in their mid-40s and older. As the disease progresses, degenerative changes occur in the synovial membrane, subchondral bone, and cartilage. Ultimately, the entire joint and its surrounding tissues become structurally and functionally impaired. Several sets of biochemical markers have been proposed to enable timely diagnosis and anticipate disease progression. However, only a few of these markers are routinely used to evaluate disease activity in subgroups. We conducted a cross-sectional, single-center cohort study of 72 patients with knee osteoarthritis. Diagnoses were established based on clinical data and radiological findings. We examined two Wnt/β-catenin signaling inhibitors, serum DKK-1 and sclerostin, and two bone/cartilage metabolic regulatory factors, RANKL and OPG, correlating these with disease activity and pain scores (WOMAC, VAS, and KOFUS), radiographic stage, inflammatory molecules and indices, and bone mineral density. DKK-1 levels were higher in the intensive pain group (VAS > 5) and positively correlated with the KOFUS throughout the study. This correlation was stronger in individuals with a BMI < 30. Serum DKK-1 levels were higher in patients with lower bone mineral density. No significant modifications in SOST, RANKL, or OPG levels were found in any of the above settings. In our patient cohort with mild-to-moderate knee osteoarthritis (OA), sclerostin, osteoprotegerin (OPG), and receptor activator of nuclear factor kappa-B ligand (RANKL) were not related to pain or disease activity. In contrast, DKK-1 was an indicator of pain and low-grade flare-ups. Furthermore, DKK-1 was associated with the KOFUS and impaired bone turnover in non-obese subgroups. Confirming these relationships in larger groups of patients would contribute to more efficient use of DKK-1 in disease stratification algorithms.

## 1. Introduction

Osteoarthritis (OA) is the most common musculoskeletal disorder and is considered a degenerative joint disease. It primarily affects individuals aged 40 and above; patients develop pain that worsens with movement, as well as stiffness and swelling. The most commonly affected joints are the knees, hips, cervical and lumbar spines, hands, and first metatarsophalangeal joints [1]. As life expectancy increases and diagnostic capabilities improve, the OA patient population continues to grow [2]. In addition to age, risk factors include sex, obesity, previous joint trauma, metabolic disease, smoking, mechanical factors, and genetic predisposition [3]. During the course of the disease, cartilage degeneration, subchondral bone remodeling, and involvement of the synovial membrane and periarticular tissue are affected to varying degrees [4], complicating the determination of relevant biomarkers.

Historical data show that OA was differentiated from rheumatoid arthritis (RA) in the early 20th century. Then, in 1950, Kellgren and Lawrence proposed the radiological scoring system that is still in use today. Knee osteoarthritis is the most common form, affecting at least half of those with OA [1]. Starting in the 1970s, multiple studies demonstrated the importance of inflammatory processes and cells in initiating and perpetuating the main pathways [5,6]. Over the last two decades, novel research has revealed growing biochemical evidence of the inflammatory process [7,8], and it has been postulated that low-grade synovitis with chronic inflammatory changes and cytokine production is present even in the early stages of OA. Indeed, IL-1 alone or in combination with a mechanical stimulus can trigger an experimental disease, and furthermore, IL-1 receptor antagonist treatment preserves cartilage by suppressing catabolic and pro-inflammatory gene expression [9,10]. However, in advanced stages before knee replacement therapy, the changes resemble those seen in rheumatoid arthritis [11].

Studies over the past two decades have demonstrated the involvement of innate immunity and tissue crosstalk between the synovium and subchondral bone. Compared to the cytokine model, this is associated with a more complex regulation of molecular events [12,13]. Endotyping OA patients as inflammatory or non-inflammatory may account for the varied treatment responses [14]. A 2021 study by Angelini et al. found that categorizing OA patients based on biochemical marker data identified three groups: low tissue turnover, structural progression, and systemic inflammation with persistent pain [15].

The co-existence of metabolic disease, low-grade inflammation, and obesity-related alterations, along with the loss of bone tissue, gives rise to the term “osteoimmunology” [16]. The osteoimmunological mechanisms of osteoporosis resemble the inflammatory pathways of osteoarthritis. Danger signal-induced inflammatory cytokines, such as TNF-α (tumor necrosis factor), IL-1β (interleukin), and IL-6, activate CD4+ T cells. This leads to the overproduction of receptor activator of nuclear factor-κB ligand (RANKL) in subchondral osteoblasts and synovial fibroblasts [17]. In a recent study using a mouse osteoarthritis model, investigators observed that RANKL-driven osteoclast activation and subchondral bone remodeling occurred before cartilage breakdown [18]. Upregulated RANKL signaling induces modifications in the subchondral and synovial joints [19], inducing bone resorption, subchondral sclerosis, and osteophyte formation. The inflammatory process is multicellular, involving T cells, neutrophil granulocytes, B cells, macrophages, mast cells, natural killer cells, plasma cells, and dendritic cells [20].

Along with the osteoprotegerin (OPG)/RANKL/RANK regulation, the Wnt/β-catenin pathway plays a crucial role in bone homeostasis and the development of osteoarthritis (OA). While it can promote joint homeostasis, it can also contribute to joint destruction [21]. When the Wnt proteins bind to cell receptors, they stabilize β-catenin. β-catenin then accumulates in the cytoplasm and translocates to the nucleus. There, it promotes the transcription of target genes of *MMPs*, *OPG*, and *RANKL* [21,22].

In OA, Wnt stimulation of osteoblasts leads to activation, subchondral sclerosis, and formation of osteophytes [23,24]. Wnt inhibitors, such as Dickkopf-1 (DKK-1) and sclerostin, help control the progression of osteoarthritis (OA) [25,26,27]. Osteocytes produce potential OA biomarkers, such as sclerostin and DKK-1, which can inhibit the canonical Wnt signaling pathway and reduce bone formation [26]. At the bone and joint level, there are multiple interactions between Wnt signaling and the OPG/RANKL regulatory pathway [28]. The canonical Wnt pathway reduces bone resorption by promoting *OPG* expression and inhibiting the expression of *RANKL*. Conversely, the non-canonical pathway may stimulate RANKL-induced osteoclastogenesis [28].

The objective of this study is to evaluate the potential role of four disease activity biomarkers, DKK-1, sclerostin, OPG, and RANKL, and their relationship with pain, flare-ups, overall disease activity, and radiographic grade in a group of knee osteoarthritis (OA) patients. Despite advances in knowledge, gaps remain in our understanding of crosstalk among signaling pathways and OA patient heterogeneity.

## 2. Results

### 2.1. Study Group Characteristics

A total of 72 patients suffering from knee osteoarthritis with an average disease duration of 3–15 years were included in the study. The patients had an average age of 68 years. Eighty-six percent were female and fourteen percent were male. None of the patients had infectious diseases, rheumatoid arthritis, other systemic autoimmune diseases, hepatitis, renal disease, or acute inflammatory syndromes. Hypertension was present in 79% of cases, heart failure in 80.5%, and diabetes in 23.6%. During the investigation, or in recent medical history, thirteen subjects presented hypothyroidism (18%), and twenty-eight (38.8%) showed signs of dyslipidemia (hypercholesterolemia, hypertriglyceridemia, or mixed). The median BMI was 25.7 (24–30.1); 53 subjects were normal or overweight (BMI < 30), whereas 19 were obese, with values ≥ 30. Twenty-one patients had decreased bone mineral density, and of these, 12 were classified as having osteopenia, while nine had osteoporosis. Concerning local symptoms, hyperemia and crepitus were the most characteristic, with mobility impairment being observed in 54.2% and overt limping in 31.9% of the study subjects. Patients had a mean Kellgren–Lawrence score of 2.35 ± 0.08, a mean WOMAC score of 25.6 ± 1.27, and VAS values of 4.41 ± 0.17. The mean KOFUS was 1.94 ± 0.30; thus, all scores indicated low to moderate disease activity.

At the beginning of the investigation, the majority of patients used topical creams and gels, analgesics, and non-steroidal anti-inflammatory drugs; 54.2% used p. o. chondroprotectors; and 26.4% had infiltrations with intra-articular corticosteroids in the previous 3 months (Table 1).

### 2.2. Principal Component Analysis

We applied principal component analysis (PCA) to identify the factors responsible for the majority of the variability. Analysis of all 49 continuous variables resulted in 21 models, 10 of which explained 82.9% of the variance. The first four models accounted for nearly 50% of the overall variance. The characteristics of these four PCs are shown in Table 2.

**Figure 1:**
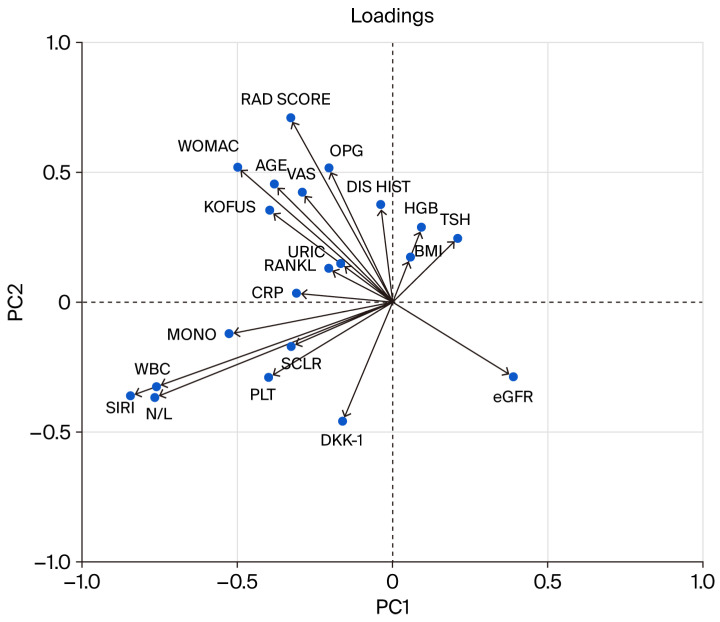
Loadings of PC1 vs. PC2. DIS HIST–disease history, RAD SCORE–radiological score, MONO–monocytes, PLT–platelets, N/L–neutrophil-to-lymphocyte ratio.

The highest eigenvalues were for PC1 (3.62) and PC2 (2.70). The loadings of these principal components are presented in Figure 1. Disease activity scores, such as WOMAC, KOFUS, the K-L grade, and VAS, all had negative coefficients in PC1 and positive coefficients in PC2. DKK-1 exerted a weak negative influence on PC1 and a stronger negative influence on PC2. WBC, neutrophil-to-lymphocyte ratio, and SIRI had negative coefficients in both.

### 2.3. Correlations of K-L, WOMAC, and KOFUS Results

To characterize the associations between radiological signs of disease severity and pain, functional scores, and biological variables, we first analyzed the correlations between the radiological grade and the aforementioned factors. The WOMAC (r = 0.539, *p* < 0.0001), VAS (r = 0.383, *p* < 0.001), and KOFUS (r = 0.303, *p* = 0.009) all showed strong positive correlations with the K-L score. Additionally, the K-L score was positively associated with hemoglobin concentration (*p* = 0.282, *p* = 0.027) and showed a tendency to correlate with serum uric acid (r = 0.243, *p* = 0.060).

Concerning pain, the VAS was strongly, positively correlated with WOMAC (<0.001), radiological grade (*p* < 0.001), and KOFUS (*p* < 0.001) in the overall group. KOFUSs were also associated with the WOMAC (*p* < 0.001) (Table 3). The VAS, KOFUS, and WOMAC were not associated with complete blood count parameters, including the white blood cell number, absolute monocyte and platelet numbers, neutrophil-to-lymphocyte ratio, systemic inflammatory response index, C-reactive protein, thyroid-stimulating hormone, and uric acid. Furthermore, VAS and WOMAC scores could not be correlated with the four elective osteoarthritis biomarkers: serum DKK-1, sclerostin, OPG, and RANKL. DKK-1 showed a significant positive association with KOFUS (r = 0.23, *p* = 0.046), while sclerostin, OPG, and RANKL were not associated with disease activity (Table 3).

**Figure 2:**
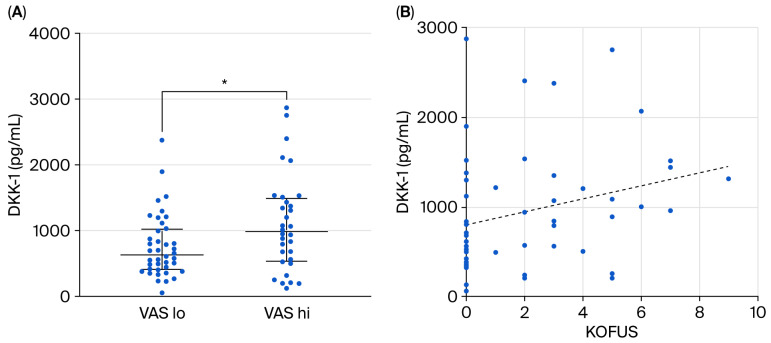
(**A**,**B**) Serum DKK-1 levels in the VAS^hi^ and the VAS^lo^ subgroups in all patients (n = 72) (**A**). Correlation of serum DKK-1 with the KOFUSs in the non-obese patients (n = 53) (**B**). * *p* = 0.040.

In the subgroup with a body mass index < 30, the correlation between serum DKK-1 levels and KOFUS was also significant, and stronger than in the whole group (r = 0.32, *p* = 0.018) (Figure 2B). If we restricted the analysis to those with BMI < 30 and normal BMD (n = 38), this correlation disappears. In this subgroup, serum DKK-1 titers showed no correlations with the K-L score (r = −0.025, *p* = 0.879), VAS (r = 0.118, *p* = 0.486), KOFUSs (r = 0.238, *p* = 0.154), or the WOMAC score (r = 0.233, *p* = 0.165).

Despite the lack of a correlation, if we divided the study subjects into VAS^lo^ (VAS < 5, n = 40) and VAS^hi^ (VAS ≥ 5, n = 32) subgroups, serum DKK-1 was found to be significantly higher in the VAS^hi^ patients than in the VAS^lo^ subgroup (997 pg/mL vs. 641 pg/mL, *p* = 0.040) (Figure 2A).

### 2.4. Analysis of DKK-1 in the Non-Obese and Low-Bone-Mineral-Density Subgroups

As shown in Table 3, the body mass index did not correlate directly with disease activity scores. However, we assumed different metabolic relationships in obese and non-obese individuals, and we specifically analyzed the correlations of DKK-1, sclerostin, OPG, and RANKL. The occurrence of diabetes and hypothyroidism, the levels of thyroid-stimulating hormone, and serum triglycerides were similar in the subgroups with BMI < 30 and BMI ≥ 30. The serum total cholesterol was more elevated in obese individuals, but without significance (222 ± 11 mg/dL vs. 197 ± 7 mg/dL). There were no significant differences in medication with topical creams, analgesics, NSAIDs, chondroprotectors, and corticosteroidal infiltrations between the subgroups.

**Figure 3:**
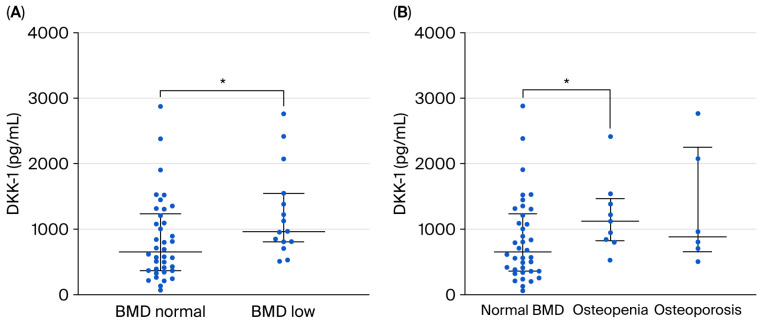
(**A**,**B**) Serum DKK-1 in patients with normal vs. abnormally low bone mineral density (**A**), normal bone mineral density vs. osteopenic vs. osteoporotic patients (**B**). * *p* = 0.020 (**A**), *p* = 0.039 (**B**).

The DKK-1 and sclerostin levels were slightly higher and OPG was lower among those with a BMI below 30, but this difference was not significant (Table 1). Among the 53 patients with normal BMI and those who were overweight, with BMI < 30, a total of 15 had an abnormally low BMD. Nine were classified as osteopenic and six as osteoporotic (28.2% together). In comparison, in the subgroup with obesity (BMI ≥ 30), 3 out of 19 subjects (15.8%) had osteopenia and osteoporosis, respectively; the difference in distribution was not significant. Thirty-eight patients with BMI < 30 and normal BMD had reduced blood levels of DKK-1, compared to the low BMD subgroup (n = 15), as shown in Figure 3A (medians 652 pg/mL vs. 965 pg/mL, *p* = 0.020). Furthermore, the normal BMD subgroup had lower values than the nine patients with osteopenia (medians 652 pg/mL vs. 1125 pg/mL, *p* = 0.039) (Figure 3B). DKK-1 levels did not differ significantly in those with osteoporosis, but this subgroup consisted of a smaller number of subjects.

### 2.5. Simple and Multiple Linear Regression

We investigated whether serum DKK-1 is a predictor of VAS and KOFUSs in simple linear regression models. In the overall patient group (n = 72), DKK-1 was not a significant determinant of either score (*p* = 0.191 for VAS and *p* = 0.112 for KOFUS). DKK-1 was not a predictor in the non-obese patient subgroup (n = 53) for the VAS (*p* = 0.156). However, DKK-1 levels were significant in predicting the KOFUSs in this subgroup (*p* = 0.046). Furthermore, we constructed multiple linear regression models to examine the impact of covariates on this relationship. Taking into account the Kellgren–Lawrence (K-L) radiological score and serum DKK-1 for analysis, adjusting for body mass index (BMI) and bone mineral density (BMD) category, a significant model was obtained (Table 4). BMI, the K-L score, and DKK-1 levels were introduced as continuous variables, while BMD was represented as a categorical variable (normal, osteopenic, or osteoarthritic). The radiological score was the stronger predictor, and DKK-1 remained a significant determinant of KOFUS.

## 3. Discussion

There is no simple correlation between pain and structural deterioration or radiological signs in knee osteoarthritis. Both the synovium and the subchondral bone, along with the bone marrow, contribute to the development of pain [28,29]. This process involves the delivery of pro-inflammatory cytokines and nerve growth factor (NGF), remodeling with continuous production of danger-associated molecular patterns, malfunction of the dorsal root ganglia’s nociceptive neurons, and neuroinflammation. Therefore, it is important to identify patients with low pain scores, reversible extracellular matrix reorganization, and low-to-medium grade cartilage destruction.

### 3.1. Correlations of Radiological Grade, Pain, and Low Osteoarthritis Flare-Up Scores

Many research studies have highlighted the complexity of the cellular and mechanical factors that contribute to the development and maintenance of low-grade inflammation and cytokine modulation [7,24]. According to theoretical considerations and experimental observations, Wnt signaling inhibitors and the OPG-RANKL regulatory axis are functionally linked to the mainstream processes of osteoarthritis. In this study, we demonstrated the importance of DKK-1 as a serum biomarker for mild-to-moderate radiologically graded knee osteoarthritis accompanied by moderate pain and no overt flare-ups. Pain assessment is subjective and depends on personal experience; it is also often influenced by emotional dysregulation and lacks a global evaluation standard [30]. We used two different pain scores: first, the visual analog scale (VAS), which ranges from 0 to 10; second, the Knee Osteoarthritis Flare-Ups Score (KOFUS) validated by Marty et al. in 2009, to understand the mutual impact of regulatory factors, pain, and joint deterioration [31,32]. The KOFUS is based on the presence of the following criteria in the knee joint: morning stiffness, swelling, limping, night pain that awakens the patient, and increased warmth over the knee. A cut-off value of 7 demonstrated excellent sensitivity (87%) and specificity (87.9%) for OA, as well as high positive and negative predictive values [32]. However, there are no data on associations with low KOFUSs. Scores of 7 or less characterized patients in only 12.9% of cases at the rheumatologists’ appointments [32]. A score above seven out of a possible 14 indicates reactivation of knee osteoarthritis (OA). Parry et al.’s longitudinal data collection revealed a significant number of patients with flare-ups primarily attributed to high physical activity [33]. In our cohort, subjects predominantly suffered from mild-to-moderate OA. The mean Kellgren–Lawrence (K-L) score was 2.35 ± 0.08; the mean Western Ontario and McMaster Universities Osteoarthritis Index (WOMAC) score was 25.6 ± 1.27; and the mean Visual Analogue Scale (VAS) score was 4.41 ± 0.17. Anti-inflammatory treatments, especially corticosteroid infiltrations, could reduce pain scores, even if distributed among subgroups without significant bias. Significantly higher DKK-1 levels were observed in those with higher VAS. The mean KOFUS was 1.94 ± 0.30. There were strong correlations between pain scores and radiological stage despite lower visual analog scale and flare-up scores than radiologically assessed joint deterioration. These associations qualify the VAS and the KOFUS as a valid and sensitive tool for detecting stealthy, small-scale underlying processes and joint deterioration pathways.

### 3.2. The Influence of an Altered Body Mass Index on Osteoarthritis Pathways

A higher body weight may influence osteoarthritic pathways in several ways: a. it charges mechanical loading; b. alters the body, and implicitly, the joint tissue composition; c. adipose tissue secretes pro-inflammatory adipokines, maintaining low-grade inflammation in the joint; d. alters insulin homeostasis; e. tunes up the immune system in a high-level alertness; f. triggers fibrosis within and around the joints [34,35,36]. Each additional pound of weight increases the load on the knee by up to four-fold. The frequently associated dyslipidemia also provokes inflammation, higher matrix metalloproteinase (MMP) and a disintegrin and metalloproteinase with thrombospondin motif (ADAMTS) activities, higher oxLDL levels, and increased oxidative stress [34]. The pathogen-associated molecular pattern (PAMP) leakage from the gut leads to more intensive Toll-like receptor triggering; activates M1-type pro-inflammatory macrophages, dendritic cells, Th17 helper lymphocytes; and is often associated with mitochondrial dysfunction [35]. A positron emission tomography tracer, along with ^18^F-sodium fluoride and ^18^fluodeoxyglucose experiments, revealed strong correlations between body mass index, bone turnover, and glucose uptake, respectively [37]. To date, the roles of DKK-1, sclerostin, OPG, and RANKL in OA patients with altered BMI are poorly understood and lack direct clinical correlations.

In our cohort, the radiological and WOMAC scores were comparable, while the VAS and the KOFUSs showed mild differences in favor of the BMI < 30 subgroup; diabetes and hypothyroidism had a similar incidence; the occurrence rate of dyslipidemia and total cholesterol was lower, but all differences lacked significance. In the non-obese, DKK-1 and sclerostin were non-significantly reduced, whereas OPG was slightly elevated; however, DKK-1 showed a significant positive correlation with the KOFUS. Since several studies have confirmed a downward trend in serum DKK-1 levels in advanced osteoarthritis (OA), our findings may represent a biphasic variation in the biomarker that increases in the low domain in parallel with the flare-up scores. Furthermore, higher OPG levels may be associated with more advanced stages of the disease; these observations could be important if confirmed in larger populations.

### 3.3. The Influence of Underlying Altered Bone Metabolism on Markers of Osteoarthritis

At the mechanistic level, the Wnt pathway and its inhibitors affect osteoarthritis and osteoporosis in opposite ways. Corrado et al. cultured osteoarthritic, osteoporotic, and healthy human primary osteoblasts and observed a decreased RANKL/OPG ratio in osteoarthritis (OA) and an increased ratio in osteoporosis (OP), relative to healthy osteoblasts [38]. In Col1a1/DKK-1 transgenic mice that have undergone meniscectomy, osteoarthritis scores improve, osteophytes become less prevalent, and subchondral bone mass decreases [39]. Denosumab treatment that suppresses RANKL and lasts 36 months in osteoporotic postmenopausal women caused a continuous decline in DKK-1 levels and an increase in sclerostin levels [40]. Butler et al. reported that osteoporotic women had serum DKK-1 levels that were nearly 70% higher than those of healthy women. Serum levels were negatively correlated with lumbar and femoral T- and Z-scores [41]. Tian et al. confirmed the significant negative correlation with bone mineral density and positive correlation with the bone catabolism marker CTX-I in a larger cohort [42]. Bone turnover is a dominant source of circulating DKK-1 and likely reflects bone–cartilage crosstalk, too. Our results reveal that bone turnover and bone mineral density may mask the secretory profiles of underlying osteoarthritis due to the dominating tissue mass, as evidenced by significantly increased serum DKK-1 titers in those with osteopenia and osteoporosis.

### 3.4. Serum DKK-1 Changes and Comparisons with Other Clinical Research Findings

Our findings revealed a slight, but significant, positive association between DKK-1 and the KOFUS in the overall patient group. Furthermore, after adjusting for bone mineral density and body mass index in a linear regression model, DKK-1 remained a determinant of the KOFUS, along with the K-L score. Among non-obese subjects with a normal or overweight BMI (<30), the KOFUS showed a stronger association, which suggests that excess body weight may obscure the effect of Wnt-related biochemical signals and highlights the importance of patient stratification as essential for biomarker interpretation. Patients with low bone mineral density (encompassing those with osteopenia and overt osteoporosis) exhibited significantly higher serum DKK-1 concentrations compared to subjects with normal BMD.

Our study indicates a link between elevated DKK-1 levels and VAS pain scores above five on a ten-point scale. The higher DKK-1 levels in those with VAS ≥ 5 DKK-1 show that DKK-1 is associated with clinically meaningful pain intensity. This association suggests a pain-linked, Wnt-inhibitory biochemical phenotype.

We hypothesized that the underlying pathways may differ between obese and non-obese patients and analyzed the latter subgroup separately. Indeed, several relationships were characteristic only of the non-obese OA subjects. These patients had slightly higher VAS and KOFUSs, as well as higher DKK-1 and sclerostin values and lower OPG serum concentrations, though none of the differences were significant.

Studies have revealed conflicting results regarding DKK-1 serum or synovial concentrations in patients with osteoarthritis of the knee compared to healthy individuals. Some authors described elevated values, but most have reported diminished values in advanced radiological disease. Theologis et al. noticed higher serum DKK-1 and comparable levels of synovial DKK-1 in OA patients in relation to non-osteoarthritic controls, and increased concentrations in the subgroup with a K-L score of 4 compared to K-L scores 2 and 3. The study subjects possessed an average age of 70 years and a BMI of 27.7 ± 3.5. They also reported that synovial DKK-1 correlates negatively with the Knee Injury and Osteoarthritis Outcome Score (KOOS) [43,44]. Our observations are in partial concordance with those of Azzam’s research from 2024, which revealed higher levels of DKK-1 in patients with osteoarthritis (OA) compared to individuals without OA, along with decreasing levels with progression of the disease [45]. In this study, serum DKK-1 had a significant inverse correlation with the radiological grades and was lower in those with ultrasonographic effusion. The population included had an average age of 53.4 ± 4 years, a higher BMI (30.4) and VAS (5.9), and much higher WOMAC scores than our cohort (60 ± 11.4) [45]. Ibrahim et al. found higher DKK-1 in OA than in healthy individuals, but lower than in rheumatoid arthritis, both in serum and synovial fluid. This study also highlighted decreased DKK-1 in advanced radiological grades (3–4). Patients were younger (51.4 ± 5.8 years), with a higher BMI (32 ± 3.8), WOMAC (54.6 ± 18) and a KOFUS that was close to the pre-established cut-off (6.8) [46]. In contrast, several other studies also revealed decreased DKK-1 in osteoarthritis compared to healthy subjects. The study by Honsawek included the analysis of synovial and plasma levels of DKK-1 in knee OA and showed a strong negative correlation with radiographic severity. The study group found synovial fluid DKK-1 levels lower than in healthy individuals, suggesting reduced systemic production of DKK-1 in OA [47]. This OA group, aged 68.8 ± 8 years, had BMI values close to those of our cohorts [47]. Hassan et al. described diminished synovial levels of DKK-1 in association with radiological progression, but no significant differences between OA patients and controls. All these studies enrolled a relatively small number of osteoarthritic subjects (a maximum of 45) [48]. In a larger case–control study (148 patients and 101 controls), Min et al. observed a reduction in DKK-1 in OA compared to the control group, setting a sensitivity of 78.6% and a specificity of 40% in the prediction of severe OA [49]. In our cohort, mild osteoarthritic cases with a Kellgren–Lawrence score of 2 had also relatively higher circulating DKK-1 than patients with advanced scores (3–4).

### 3.5. Osteoprotegerin and RANKL

Osteoprotegerin and RANKL are important regulatory factors in both bone and joint homeostasis. OPG, a decoy receptor of RANKL, blocks osteoclastogenesis and cell maturation. OPG also controls the Wnt/β-catenin pathway [28,50]. Two types of osteoarthritic osteoblasts have been identified: low-level and high-level synthesizers. The former produces less OPG and more RANKL, while the latter expresses higher levels of OPG and shows an increased OPG/RANKL ratio. In osteoarthritic cartilage, catabolic and senescent chondrocytes also produce less OPG and higher levels of RANKL in a pro-inflammatory microenvironment [28]. Serum OPG and RANKL levels were higher in knee OA patients than in controls; synovial OPG concentrations were almost four times higher than serum levels [51]. An early study reported elevated serum and synovial OPG levels in OA but not in rheumatoid arthritis or spondyloarthritis [52]. Quaresma et al. found that synovial levels of OPG and RANKL could not differentiate between rheumatoid arthritis, spondyloarthritis, and osteoarthritis. However, the RANKL/OPG ratio in the osteoarthritis group showed a strong positive association with C-reactive protein levels [53]. In a previous study, when we compared small groups of patients with early- and advanced-stage OA, we failed to reveal differences in serum and synovial OPG levels [54]. Several factors may generate relevant fluctuations of this marker. Many tissues, such as bone, epithelium, and endothelium, produce OPG. Circulating concentrations are influenced by protein glycosylation and AB0 blood groups [55]. Inflammatory cell degradative enzymes, such as neutrophil elastase, cleave OPG [56]; however, the importance of this interaction in vivo needs further proof [57]. In the current study, OPG levels were slightly higher in obese patients but did not correlate with the WOMAC, VAS, or KOFUSs. RANKL serum levels showed minimal variations, no difference between the BMI-classified groups, and no association with the WOMAC, VAS, and KOFUS.

### 3.6. Serum Sclerostin

Sclerostin controls osteoblast proliferation. It preserves chondrocyte metabolism by controlling the canonical and non-canonical Wnt pathways. Furthermore, knocking out the *SOST* gene in mice has a detrimental effect on cartilage [58]. In contrast, *SOST* transgenic mice developed only mild post-traumatic osteoarthritis (OA) alongside reduced MMP2 and MMP3 protein levels [59]. Eldin et al. studied 50 OA patients and 30 healthy individuals and found significantly lower plasma sclerostin levels in patients, as well as significant inverse correlations with physical disability, disease duration, and radiological severity [60]. In contrast, in the OFELY study, serum sclerostin levels were not associated with Kellgren–Lawrence scores or radiological progression over a four-year follow-up period [61].

A Polish study of acromegaly patients examined the relationship between OPG/RANKL and SOST in bone resorption and bone mineral density. The study revealed that SOST modulates OPG/RANKL in a compensatory manner [62].

However, our study found no significant association between sclerostin and pain or radiological scores. Specifically, sclerostin could not be associated with disease activity (WOMAC), pain scores (VAS and KOFUS), or radiological deterioration (Kellgren–Lawrence) in the entire cohort or the non-obese subgroup. Alterations in bone mineral density and microarchitecture induced by osteopenia and osteoporosis are linked to multi-omics (proteomics, metabolomics, and genomics) and multiple regulatory mechanisms, including oxidative stress, chronic inflammation, and gut microbiota [63]. The relatively small sample size, consisting mainly of patients with mild-to-moderate disease, may explain this result.

### 3.7. Relevance of the Study

The current observational study focused on four biomarkers: the Wnt pathway inhibitors DKK-1 and sclerostin, and the proteins osteoprotegerin and RANKL. Under certain conditions, these biomarkers reflect pathological changes characteristic of knee osteoarthritis and may serve as therapeutic targets. There is an upward trend in trials involving disease-modifying osteoarthritis drugs (DMOADs) targeting these pathways. For example, SM04690 (lorecivin) is a promising molecule that aims to modulate the Wnt/β-catenin pathway to prevent or slow disease progression [64]. Subgroup analysis was a secondary goal of this study. The subgroups were not pre-designed; rather, we used the natural distribution of the data for their stratification. Although the observed between-group effect sizes were in the small-to-moderate range, this magnitude is consistent with established osteoarthritis interventions and exceeds commonly accepted minimal clinically important differences for pain and function outcomes. Further research is needed to confirm the relevance of DKK-1 changes, focusing on joint structure deterioration and bone mineral density loss.

### 3.8. Interactions and Limitations

There are likely many more complex interactions among different pathways, as well as between osteoarthritis (OA) phenotypes, individual patient risk factors, comorbidities like osteoporosis or metabolic disease, and triggering events that contribute to the development and progression of the disease. Our study had a cross-sectional design, lacking a control population, and recruitment was limited to a single center. We did not have synovial fluid samples to investigate, which could have provided additional useful information. Because patient selection for this study was random and not based on body mass index or bone mineral density, the subgroups were imbalanced. Another limitation is that the KOFUSs were predominantly in the inferior range. Thus, the results cannot be directly applied to overt osteoarthritic flare-ups and should be confirmed in larger populations using a broader KOFUS scale.

## 4. Materials and Methods

### 4.1. Study Design and Patient Cohort

Between June 2023 and June 2025, we examined a total of 72 patients with primary knee osteoarthritis (62 females and 10 males; mean age: 68 years). All subjects were recruited from the Procardia Outpatient Medical Service in Târgu Mureș and were suffering from knee osteoarthritis at various radiographic stages. Patients with acute inflammatory diseases, severe liver disease, infections, systemic autoimmune disease, or neoplastic disorders were excluded from the study.

Each subject was informed about the study and asked to sign an informed consent form. The study was approved by the Ethics Committee (approval no. 01/4 June 2023). We took a thorough medical history and performed a physical examination in each case. We documented the patients’ demographic information, as well as details regarding disease manifestation, professional risk factors, and medical treatment received. We also noted the presence of any comorbidities. Patients were evaluated in the context of disease activity using three scoring systems: the Visual Analogue Scale (VAS) for pain, the Knee Osteoarthritis Flare-up Score (KOFUS) for OA flare-ups, and the Western Ontario and McMaster Universities Osteoarthritis Index (WOMAC) for pain, stiffness, and daily activities.

### 4.2. Laboratory Testing

Venous blood samples were collected into Vacutainer tubes with no additive, EDTA-K3, or 3.2% trisodium citrate (Becton–Dickinson Vacutainer Systems, Wokingham, UK) after overnight fasting. Tubes intended for biochemical and coagulation testing were centrifuged at 3000 rpm for 10 min to separate the serum and plasma. C-reactive protein, glucose, creatinine, transaminases, uric acid, total cholesterol, and triglycerides were determined from these samples using a Mindray BS480 analyzer (Shenzhen, China); concentrations of thyroid-stimulating hormone were measured using a Mindray CL900i, and plasma fibrinogen was determined using a Coag M analyzer (Diatron, Budapest, Hungary). The complete blood count was determined from the EDTA-K_3_ tubes using a Mindray 5380 analyzer (Shenzhen, China), and the neutrophil-to-lymphocyte ratio (N/L) and systemic inflammatory response index (SIRI) were calculated.

### 4.3. Determination of Disease Activity Biomarkers

Two serum specimens were frozen at −20 °C after centrifugation for subsequent analysis of the specific biomarkers OPG, RANKL, DKK-1, and sclerostin. We used commercially available ELISA kits to analyze the serum concentrations of OPG (R&D Systems, Minneapolis, MN, USA, Human Osteoprotegerin/TNFRSF11B Duoset ELISA, DY805), RANKL (R&D Systems, Human TRANCE/RANKL/TNFSF11 Duoset ELISA, DY626), sclerostin (R&D Systems, Human SOST/Sclerostin Duoset ELISA, DY1406), and DKK-1 (R&D Systems, Human DKK-1 Duoset ELISA, DY1906). The complementary reagents were from the Duoset ELISA Ancillary Reagent Kit 2, and the reactions were read using the automated ThunderBolt instrument (Gold Standard Diagnostics, Horsham, PA, USA). The manufacturer’s recommendations were followed when performing these determinations.

### 4.4. Radiographic Examination

An X-ray of the knee was taken, including a comparative orthostatic view and a lateral view of the symptomatic knee. Radiographic staging was performed using the Kellgren–Lawrence classification system, which categorizes osteoarthritis into four grades: grade 1, suspicious joint space reduction; grade 2, definite osteophytes and probable joint space narrowing; grade 3, moderate osteophytes, definite joint space narrowing, and subchondral sclerosis; and grade 4, large osteophytes, marked joint space narrowing, severe subchondral sclerosis, and bone deformities.

Bone mineral density was determined using dual X-ray absorptiometry (DEXA) on the lumbar spine in a different outpatient medical service. T-scores between 1.5 and 2.49 were considered moderate-to-advanced osteopenia, while values below −2.5 were classified as osteoporosis.

### 4.5. Statistical Analysis

We analyzed the data distribution using the Lilliefors and Shapiro–Wilk tests. We presented the results according to the distribution type: mean ± standard error (SE) or median (quartile range). We performed a general data exploration using principal component analysis to calculate the eigenvalues and proportion of variance for the main PCs. Considering the group sizes, we used the Mann–Whitney U test for between-group comparisons and Spearman’s rank correlation analysis. No corrections were applied for multiple testing. We constructed a multiple linear regression model to determine the effect of the variables on the VAS and KOFUS in the entire group. We processed the data using GraphPad Prism 10.6.1 and Microsoft Excel 2016. We set the significance threshold at *p* = 0.05.

## 5. Conclusions

This study demonstrated the potential benefits of evaluating serum DKK1 levels in patients with knee osteoarthritis. We highlighted that elevated DKK-1 levels were associated with higher pain scores and KOFUSs in mild-to-moderate disease stages, whereas sclerostin, OPG, and RANKL were not related to any of the clinical activity measures. These results should be interpreted taking into consideration the metabolic background, body mass index, and impaired bone turnover. Our findings suggest that DKK-1 is a context-dependent indicator of pain and low-grade flare activity, with clinical relevance in specific patient subgroups defined by body mass index and bone metabolic status. Given the episodic nature of flare-ups, validation in further studies, ideally including longitudinal assessment, is necessary.

## Figures and Tables

**Table 1:** Clinical variables of the overall group of OA patients, and their comparison across BMI subgroups.

Variable	All Cases (n = 72)	BMI < 30 (n = 53)	BMI ≥ 30 (n = 19)	*p* Values
Demographic factors				
Age (years)	68 (63–75)	68 (62–75)	68 (63–75)	0.829
Gender (f/m)	62 (86.1)/10 (13.9)	44 (83)/9 (17)	18 (94.4)/1 (5.6)	0.272
History, symptoms, and comorbidities				
Disease history (years)	7 (3–15)	7 (3–11)	10 (5–15)	0.120
Crepitus (y/n)	50 (69.4)/22 (30.6)	34 (64.2)/19 (35.8)	16 (84.2)/3 (15.8)	0.148
Sensibility (y/n)	27 (37.5)/45 (62.5)	17 (32.1)/36 (67.9)	10 (52.6)/9 (47.4)	0.166
Hyperemia (y/n)	62 (86.1)/10 (13.9)	45 (84.9)/8 (15.1)	17 (89.5)/2 (10.5)	1.000
Swelling (y/n)	7 (9.7)/65 (90.3)	5 (9.4)/48 (90.6)	2 (10.5)/17 (89.5)	0.988
Bony prominences (y/n)	17 (23.6)/55 (76.4)	12 (22.6)/41 (77.4)	5 (35.5)/14 (64.5)	0.759
Mobility impairment (y/n)	39 (54.2)/33 (45.8)	26 (49)/27 (51)	13 (68.4)/6 (31.6)	0.184
Coxalgia (y/n)	6 (8.3)/67 (91.7)	3 (5.6)/50 (94.4)	3 (15.8)/16 (84.2)	0.183
Dysplasia (y/n)	5 (6.9)/67 (93.1)	5 (9.4)/48 (90.6)	0 (0)/19 (100)	0.316
Limping (y/n)	23 (31.9)/49 (68.1)	15 (28.3)/38 (71.7)	8 (42.1)/11 (57.9)	0.389
Previous trauma (y/n)	6 (8.3)/67 (91.7)	4 (7.5)/49 (92.5)	2 (10.5)/17 (89.5)	0.647
Heart failure (y/n)	58 (80.5)/14 (19.5)	41 (77.5)/12 (22.5)	17 (89.5)/2 (10.5)	0.714
Hypertension (y/n)	57 (79.2)/15 (20.8)	41 (77.5)/12 (22.5)	16 (84.2)/3 (15.8)	0.744
Diabetes (y/n)	17 (23.6)/55 (76.4)	13 (24.5)/38 (75.5)	4 (23.5)/17 (76.5)	0.761
Hypothyroidism (y/n)	13 (18)/59 (82)	9 (17)/44 (83)	4 (21)/15 (79)	0.692
Dyslipidemia (y/n)	28 (38.8)/54 (61.2)	18 (33.9)/35 (66.1)	10 (52.6)/9 (47.4)	0.152
Osteopenia/osteoporosis (y/n)	12 (16.6)/9 (12.5)/51 (70.9)	9 (16.9)/6 (11.3)/38 (71.8)	3 (15.7)/3 (15.7)/13 (68.6)	0.600
Clinical data				
Body mass index	25.7 (24–30.1)	24.5 (23.7–26.6)	31 (30.4–31.6)	<0.001
Kellgren–Lawrence score *	2.35 ± 0.08	2.30 ± 0.09	2.50 ± 0.14	0.328
WOMAC *	25.6 ± 1.27	25.5 ± 1.42	26.2 ± 2.83	0.761
VAS *	4.41 ± 0.17	4.51 ± 0.20	4.16 ± 0.31	0.274
KOFUS *	1.94 ± 0.30	2.02 ± 0.34	1.73 ± 0.65	0.518
Laboratory findings				
C-reactive protein (mg/L)	3.2 (1.8–5.2)	3.2 (1.6–4.8)	3.8 (2.8–7.1)	0.064
White blood cells (10^9^/L)	6.4 (5.6–7.3)	6.3 (5.6–7.2)	7.1 (5.7–7.8)	0.561
Hemoglobin (g/dL)	13.6 (13–14.4)	13.6 (12.9–14.4)	13.8 (13.4–14.4)	0.627
N/L ratio	1.9 (1.4–2.4)	1.9 (1.3–2.3)	1.9 (1.4–2.4)	0.638
Monocytes (10^9^/L)	400 (330–510)	395 (330–500)	450 (300–560)	0.793
SIRI	705 (519–1188)	6898 (533–880)	945 (466–1398)	0.498
Uric acid (mg/dL)	4.9 (4.3–5.7)	4.8 (4.4–5.8)	5.5 (4.2–5.6)	0.558
eGFR (mL/min/1.73 m^2^) *	82.9 ± 2.1	80.0 ± 2.5	80.9 ± 3.9	0.963
Cholesterol, total (mg/dL) *	204 ± 6	197 ± 7	222 ± 11	0.197
Triglycerides (mg/dL) *	147 ± 9	147 ± 11	143 ± 14	0.711
TSH (µUI/mL)	1.9 (1.2–3.3)	1.9 (1.2–3.1)	2.0 (1.5–3.4)	0.724
DKK-1 (pg/mL)	802 (467–1234)	808 (497–1306)	693 (456–1207)	0.667
Sclerostin (pg/mL)	104 (59–526)	115 (59–535)	76 (55–328)	0.713
OPG (pg/mL)	3344 (2599–4654)	2904 (2222–4073)	3669 (2698–5156)	0.462
RANKL (pg/mL)	61 (43–368)	58 (41–428)	61 (43–244)	0.830
Medication				
Topical creams and gels	68 (94.4)/4 (5.6)	49 (92.4)/4 (7.6)	19 (100)/0 (0)	0.221
Analgesics	42 (58.3)/30 (41.7)	32 (67.9)/21 (32.1)	10 (52.6)/9 (47.4)	0.559
NSAIDs, p.o.	51 (70.8)/21 (29.2)	37 (69.8)/16 (30.2)	14 (73.6)/5 (26.4)	0.751
Chondroprotectors, p.o.	39 (54.2)/33 (45.8)	29 (54.7)/24 (45.3)	10 (52.6)/9 (47.4)	0.876
Corticosteroids, local infiltrations	19 (26.4)/53 (73.6)	13 (24.5)/40 (75.5)	6 (31.6)/13 (68.4)	0.552

Variables with normal distribution (marked with an asterisk) are represented by mean ± standard error (SE), while variables with abnormal distribution are shown as median (quartile range). For categorical variables, comparisons were made using Fisher’s exact test. The BMI groups were compared using the Mann–Whitney U test for continuous variables. *p* values less than 0.05 were considered statistically significant.

**Table 2:** Principal component analysis defining PCs 1–4.

PC Summary	PC1	PC2	PC3	PC4
Eigenvalue	3.62	2.70	2.11	1.85
Proportion of variance	17.24%	12.87%	10.07%	8.81%
Cumulative proportion of variance	17.24%	30.12%	40.19%	48.99%

**Table 3:** Correlations between the VAS and the KOFUSs in the overall group (n = 72).

	VAS		KOFUS		WOMAC	
Variables	Spearman R	*p*-Level	Spearman R	*p*-Level	Spearman R	*p*-Level
Age	0.123	0.302	0.233	0.048	0.297	0.011
Disease history	0.082	0.491	0.025	0.834	0.278	0.018
Body mass index	0.071	0.550	−0.026	0.826	0.111	0.353
WOMAC	0.651	<0.001	0.534	<0.001	-	-
K-L score	0.430	<0.001	0.303	0.009	0.539	<0.001
VAS	-	-	0.597	<0.001	0.623	<0.001
KOFUS	0.597	<0.001	-	-	0.534	<0.001
WBC	0.134	0.303	0.087	0.507	0.082	0.529
N/L ratio	0.023	0.855	−0.048	0.714	0.085	0.514
Monocytes	0.097	0.454	−0.034	0.794	0.101	0.438
SIRI	0.099	0.445	−0.042	0.746	0.147	0.257
Hemoglobin	0.224	0.081	0.034	0.794	0.053	0.680
eGFR	0.010	0.937	−0.087	0.504	−0.089	0.489
Platelets	0.163	0.210	−0.026	0.840	0.229	0.075
C-reactive protein	0.139	0.242	−0.017	0.885	0.203	0.087
TSH	−0.214	0.102	0.036	0.787	0.101	0.445
Uric acid	0.016	0.901	−0.042	0.750	−0.114	0.385
DKK-1	0.166	0.191	0.236	0.046	0.147	0.215
Sclerostin	0.000	0.999	0.102	0.394	−0.093	0.434
OPG	0.003	0.977	0.040	0.740	0.100	0.402
RANKL	0.083	0.750	0.004	0.971	0.059	0.621
OPG/RANKL	0.071	0.550	0.065	0.588	0.023	0.844

Correlations were calculated with the Spearman rank correlation method. *p* < 0.05 has been considered the threshold of statistical significance.

**Table 4:** Multiple linear regression model for the prediction of the KOFUSs.

Variable	Estimate	95% CI	t	*p* Value
Intercept	−3.500	−7.204 to 0.204	1.90	0.063
Body mass index	0.060	−0.061 to 0.181	0.99	0.322
Bone mineral density	0.256	−0.708 to 1.220	0.53	0.596
Kellgren–Lawrence score	1.311	0.413 to 2.209	2.94	0.005
sDKK-1	0.001	4.413 × 10^−7^ to 0.002	2.01	0.049

F = 3.74 and *p* = 0.01 for the overall regression model.

## Data Availability

The data presented in this study are openly available as: Nagy, Előd Ernő; Nagy-Finna, Csilla (2026), “Knee_OA_72_data_2026”, Mendeley Data, V1, DOI: 10.17632/m5b9hfbkk8.2.

## Abbreviations

The following abbreviations are used in this manuscript:

ADAMTS	A disintegrin and metalloproteinase with thrombospondin motifs
BMI	Body mass index
BMD	Bone mineral density
DKK-1	Dickkopf-related protein 1
DMOADs	Disease-modifying osteoarthritis drugs
IL-1	Interleukin-1
KOFUS	Knee osteoarthritis flare-up score
MMP	Matrix metalloproteinase
NSAIDs	Non-steroidal anti-inflammatory drugs
OA	Osteoarthritis
OPG	Osteoprotegerin
RA	Rheumatoid arthritis
RANK	Receptor activator of nuclear factor κB
RANKL	Receptor activator of nuclear factor κB ligand
SOST	Sclerostin
TNF	Tumor necrosis factor
VAS	Visual analogue scale
WOMAC score	Western Ontario and McMaster University score
